# Development of the basal hypothalamus through anisotropic growth

**DOI:** 10.1111/jne.12727

**Published:** 2019-05-23

**Authors:** Travis Fu, Caroline Pearson, Matthew Towers, Marysia Placzek

**Affiliations:** ^1^ Department of Biomedical Science Bateson Centre University of Sheffield Sheffield UK

**Keywords:** anisotropic growth, development, Fgf10, hypothalamus, prechordal mesendoderm, progenitor, sonic hedgehog

## Abstract

The adult hypothalamus is subdivided into distinct domains: pre‐optic, anterior, tuberal and mammillary. Each domain harbours an array of neurones that act together to regulate homeostasis. The embryonic origins and the development of hypothalamic neurones, however, remain enigmatic. Here, we summarise recent studies in model organisms that challenge current views of hypothalamic development, which traditionally have attempted to map adult domains to correspondingly located embryonic domains. Instead, new studies indicate that hypothalamic neurones arise from progenitor cells that undergo anisotropic growth, expanding to a greater extent than other progenitors, and grow in different dimensions. We describe in particular how a multipotent *Shh* ^*/*^ *Fgf10*‐expressing progenitor population gives rise to progenitors throughout the basal hypothalamus that grow anisotropically and sequentially: first, a subset displaced rostrally give rise to anterior‐ventral/tuberal neuronal progenitors; then a subset displaced caudally give rise to mammillary neuronal progenitors; and, finally, a subset(s) displaced ventrally give rise to tuberal infundibular glial progenitors. As this occurs, stable populations of *Shh* ^*+ive*^ and *Fgf10* ^*+ive*^ progenitors form. We describe current understanding of the mechanisms that induce *Shh* ^*+ive*^ */Fgf10* ^*+ive*^ progenitors and begin to direct their differentiation to anterior‐ventral/tuberal neuronal progenitors, mammillary neuronal progenitors and tuberal infundibular progenitors. Taken together, these studies suggest a new model for hypothalamic development that we term the “anisotropic growth model”. We discuss the implications of the model for understanding the origins of adult hypothalamic neurones.

## 1 THE ADULT HYPOTHALAMUS: FUNCTION AND ORGANISATION

The hypothalamus is an evolutionarily‐ancient part of the ventral forebrain. Its overall organisation and resident cell types have been highly conserved in eukaryotes,^1^ reflecting the crucial role of the hypothalamus to life. It is the central autonomic regulator of homeostatic mechanisms, including energy balance, growth, stress regulation, sleep and reproduction. It integrates numerous inputs, including those from sensory afferents and circulating peripheral systems, compares these with ideal basic set‐points for features such as hormone and metabolite levels, temperature and electrolyte balance, and then initiates feedback systems to restore optimal physiology. In addition, the hypothalamus mediates allostasis, that is,  the ability to re‐evaluate optimal set‐points to anticipate the organism's changing environment. The adaptive responses of homeostasis and allostasis operate through autonomic, endocrine and behavioural systems and over different durations of time to maximise the chance of individual and species survival. In this way, hypothalamic cells enable the body to respond, anticipate and adapt to changing physiological conditions over life.

Classically, the adult hypothalamus is divided into four domains: pre‐optic, anterior, tuberal and mammillary. Each domain harbours cell clusters, termed nuclei, and less well‐defined territories, all arranged in a patchwork manner. Early reports, based on lesion studies, led to the idea that a particular nucleus, or territory, might centrally control a particular behaviour; however, sophisticated new approaches, including cell‐specific and conditional knockouts, chemogenetic and optogenetic studies, now suggest that activation of a particular neurone increases the likelihood of an event or a behaviour, such that homeostasis of a particular physiological state is mediated by complex interactions of multiple nuclei/neurones.^2^

## 2 EARLY MODELS OF HYPOTHALAMIC DEVELOPMENT

There is a pressing need to determine how particular hypothalamic neurones arise in life, to provide insight into the ability of the body to anticipate and adapt robustly, to provide insight into pathological conditions/dysfunctional behaviours such as chronic stress, reproductive and eating disorders, and to inform efforts to direct the differentiation of human pluripotent cells to hypothalamic neuronal fates, all studies with enormous potential for the evaluation of future novel therapies for conditions such as obesity. Many previous models of hypothalamic development have been proposed and two in particular have received much attention: the columnar model and the prosomeric/revised prosomeric model. Each suggests that adult domains, and their resident nuclei/territories/neurones, arise from correspondingly located embryonic domains that expand isotropically (ie, to the same extent). The columnar model suggests that the hypothalamus is a diencephalic‐derived structure, with pre‐optic, anterior, tuberal and mammillary progenitor subsets arrayed in columns along the anterior‐posterior (future rostro‐caudal) axis, reflecting an early anterior‐posterior regionalisation of the neural tube.^3^ The prosomeric/revised prosomeric model^4^, ^5^ suggests that adult domains/nuclei/neurones reflect the position of progenitors in the alar or basal plate. In this model, alar and basal territories are defined on the basis of their position relative to a diagonal stripe of cells that express the signalling molecule, Sonic hedgehog (Shh). Alar: progenitors lie rostral/superior to *Shh* ^*+ive*^ cells, and basal progenitors lie within/caudal/inferior to *Shh* ^*+ive*^ cells. Furthermore, this model suggests that alar progenitors arise from a common diencephalic/telencephalic unit. In the revised prosomeric model, both ventral and dorsal parts of the anterior hypothalamus (containing the suprachiasmatic nucleus [SCN], paraventricular nucleus [PVN] and periventricular nucleus [PeVN] respectively) are derived from alar progenitors, whereas tuberal and mammillary neurones/nuclei (including the arcuate nucleus [ARC] and ventromedial nucleus [VMN]) are derived from basal progenitors. Importantly, proponents of each model point out that these provide a useful starting point for probing the origins of hypothalamic neurones, but acknowledge the difficulties in ascribing adult neuronal populations to progenitor domains, not least because differentiating neurones may undergo complex migrations.^6^, ^7^ Each model was proposed before the advent of conditional knockout approaches, or sophisticated lineage‐tracing studies, and so neither takes account of the extensive growth of the hypothalamus in the earliest stages of its development, or of the possibility that progenitor cells might migrate as they are specified.

Recent work in the embryonic chick, which examines the growth of a previously‐undefined progenitor population, now suggests that progenitor displacement/migration is key to hypothalamic development, and suggests a fundamentally different model of hypothalamic development to those previously suggested. Here, we summarise these studies^8^ and describe an “anisotropic growth model” of hypothalamic development.

## 3 ANISOTROPIC GROWTH MODEL OF HYPOTHALAMIC DEVELOPMENT

### 3.1 Induction of *Shh* ^*+ive*^ ventral midline cells

**Figure 1:**
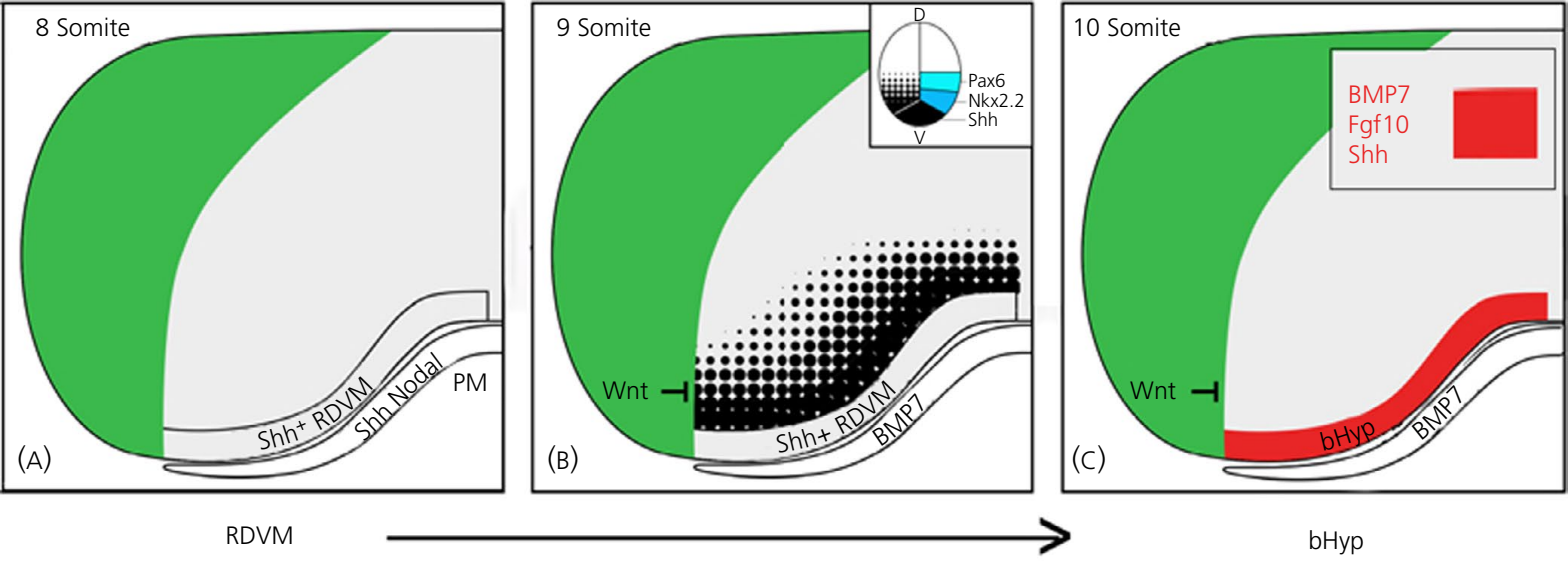
Prechordal mesendoderm induces rostral diencephalic ventral midline (RDVM)/basal hypothalamic (bHyp) cells. Schematic sagittal views of chick embryo (8‐10 somites). A, Induction of *Shh* ^*+ive*^ RDVM cells in the 8 somite embryo through Shh/Nodal from underlying prechordal mesendoderm. PM, prechordal mesendoderm. B, Establishment of dorso‐ventral pattern through a Shh morphogen gradient from RDVM cells: inset shows patterned progenitor domains. C, Differentiation to bHyp cells that co‐express *Shh*,*BMP7* and *Fgf10* (red area), under the influence of BMP7 from prechordal mesendoderm

A specialised axial cell population, the prechordal mesendoderm (PM) underlies the prospective hypothalamus for many hours in the neurula stage embryo.^9^, ^10^ Differential tissue movements, and the rapid proliferation of basal hypothalamic progenitor cells (see below), results in the PM being in register, first with the entire prospective hypothalamus, then the posterior (mammillary) hypothalamus, then the caudal diencephalon.^8^, ^10^, ^11^, ^12^ However, gain‐ and loss‐of function studies in a range of vertebrates suggest that even such transient apposition is sufficient for the PM to initiate one of the earliest steps in hypothalamic development: the induction of a population of *Shh*‐expressing ventral midline forebrain cells, termed rostral diencephalic ventral midline (RDVM) cells^13^, ^14^, ^15^, ^16^, ^17^, that extend to the boundary with Foxg1,^8^ (ie, the telencephalic boundary)^18^ (Figure 1A). As discussed below, RDVM cells play a critical role in subsequent steps in hypothalamic development.

The PM expresses the secreted glycoprotein, Shh, and studies of isolated chick tissue explants reveal that Shh is required to induce *Shh* ^*+ive*^ RDVM cells.^13^, ^15^ Other factors, however, synergise with Shh to mediate this event, including the transforming growth factor β signalling ligand, Nodal, deriving from the PM^15^, ^19^, ^20^ and the transcription factor (TF), Six3.^16^ In *Shh*‐null embryos, embryos haploinsufficient for *Six3*, or with dysfunctional Nodal signalling, RDVM cells are not induced and embryos develop holoprosencephalic phenotypes^16^, ^21^. The precise regulation and duration of *Shh* expression in the PM is crucial for RDVM cell induction. Loss of a single copy of *Shh*, or mutations that lead to reduced expression of *Shh* in the PM, result in holoprosencephaly.^22^ The temporal perturbation of Shh signalling correlates with the severity of holoprosencephalic phenotypes: the earlier the alteration, the more severe the phenotype.^23^, ^24^ The tight temporal control of *Shh* in the PM is regulated by Nodal, which acts in a juxtacrine manner to control the duration of *Shh* expression.^25^ Elegant analyses in mouse show that *Shh* expression in RDVM cells is regulated by a unique enhancer, SBE2 (Shh brain enhancer 2).^26^

Once induced, Shh diffuses out of RDVM cells to establish a morphogen gradient in adjacent diencephalic cells that is translated into a cell‐intrinsic GliA‐GliR gradient,^27^, ^28^, ^29^ similar to that found in the spinal cord.^30^ The predicted GliA‐GliR gradient is considered to set up an early dorso‐ventral pattern, characterised by domains of Shh and the homeodomain TFs, Nkx2.1, Nkx2.2 and Pax6 (Figure 1B, inset): Nkx2.2 is expressed in a diagonal stripe of cells that span the accepted basal‐alar boundary.^4^, ^5^, ^11^, ^16^, ^17^, ^27^, ^31^ Recent work validates this idea: in mice where *Shh* is deleted in RDVM cells, (Shh^Δhyp^ mice), Nkx2.2 is reduced and Pax6 expands ventrally.^29^ Cross‐repressive TF interactions may then sustain pattern: in zebrafish, triple knockdown of nkx2.1, nkx2.4a and nkx2.4b leads to the ventral expansion of pax6.^32^

### 3.2 *Shh* ^*+ive*^ RDVM cells develop into *Shh* ^*+ive*^ *Fgf10* ^*+ive*^ bHyp cells

**Figure 2:**
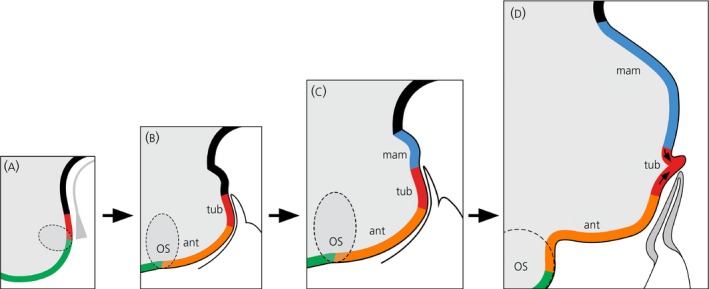
Three‐dimensional sequential anisotropic growth from basal hypothalamic (bHyp) cells. Schematic sagittal views of chick embryo (10‐40 somites). A, bHyp cells (red) abut the telencephalon (green) in the 10‐somite embryo. B, By 12 somites, bHyp cells begin to generate anterior progenitors (orange). C, By 25 somites, mammillary progenitors are generated (blue): these extend posteriorly from bHyp cells that are now central (red). D, Finally, infundibular glial cells are generated and grow ventrally (arrows). Dotted circle indicates optic stalk (os). Schematics show hypothalamus relative to underlying tissues: prechordal mesendoderm (A); or Rathke's pouch (C,D). ant, anterior; mam, mammillary; os, optic stalk; tub, tuberal

By contrast to floor plate cells at the ventral midline of the posterior neuraxis, *Shh*‐expressing RDVM cells undergo profound molecular changes: double label in situ hybridisation studies in the chick show that *Shh* ^*+ive*^ RDVM cells are the precursors to a *Shh/BMP7/Fgf10*‐expressing population that is in precise register with the underlying PM, and that*,* at its anterior end*,* abuts *Foxg1* ^*+ive*^ telencephalic progenitors^8^ (Figures 1C and 2A, red‐green). This progenitor population therefore appears to be a ventral subset of a larger diencephalic subset, potentially one analogous to the *Foxd1* ^*+ive*^ progenitor subset, which has been shown, in the mouse, to give rise to the hypothalamus and pre‐thalamus.^33^

Studies in chick and mouse indicate a likely mechanism for the transition of *Shh* ^*+ive*^ RDVM to *Shh* ^*+ive*^ */BMP7* ^*+ive*^ */Fgf10* ^*+ive*^ cells. After inducing *Shh* ^*+ive*^ RDVM cells, the PM down‐regulates *Shh/Nodal*, and up‐regulates *BMP7*, which in turn induces its own expression (and that of T‐box transcriptional repressors, *Tbx2/Tbx3,* and *BMP4*) in RDVM cells.^25^, ^34^, ^35^, ^36^ BMP, acting in a paracrine manner from the PM, or in a juxtacrine manner from RDVM cells, induces *Fgf10* in RDVM cells.^35^ We refer to cells that (transiently) co‐express *Shh/BMP/Tbx2/Fgf10* as bHyp (basal hypothalamic) cells. A number of studies indicate that the hypothalamus, including bHyp cells, can only develop if Wnt signalling is decreased.^37^, ^38^ Wnts deriving from telencephalic progenitor cells*,* may restrict the anterior limit of the hypothalamus, including the bHyp domain.^38^, ^39^, ^40^ The mechanism through which decreased Wnt signalling might support hypothalamic development is unclear, although, potentially, it enables the induction of *Tbx* genes: studies in chick demonstrate that BMP may induce Tbx2 by decreasing levels of Wnt/Wnt signalling.^35^

### 3.3 bHyp cells are proliferating progenitors that give rise to the basal hypothalamus through anisotropic growth

As RDVM cells transit to bHyp cells, they undergo pronounced changes in cell cycle: first they undergo a transient arrest, then they become highly proliferative.

Targeted DiI/DiO fate‐mapping studies in the chick embryo show the fate of the proliferative bHyp progenitor cells. They give rise to other progenitor subtypes that, through growth/displacement, extend throughout the basal hypothalamus from the optic vesicle to the mammillary pouch.^8^ Growth of particular progenitor subsets occurs anisotropically and sequentially from bHyp cells: first a subset is displaced/migrates rostrally and gives rise to *Six3* ^*+ive*^ *Foxg1* ^*−ive*^ *Fgf10* ^*−ive*^ “anterior” progenitors (Figure 2B, orange), then a subset is displaced/migrates caudally and gives rise to *Emx2* ^*+ive*^ mammillary progenitors (Figure 2C, blue); finally, progenitor(s) are displaced ventrally and give rise to tuberal infundibular progenitors^41^, ^42^ (Figure 2D, arrows). As anterior progenitors are generated, the bHyp domain resolves into distinct domains of *Shh* ^*+ive*^ and *Fgf10* ^*+ive*^ progenitors: each of these is then stably‐maintained throughout embryogenesis. Indeed, a pool of undifferentiated *Fgf10* ^*+ive*^ progenitors appears to be retained throughout life (beyond the scope of the present review; Placzek M., Fu T., Towers M. [submitted]). The sequential anisotropic growth in three‐dimensions from bHyp progenitor cells is peculiar and unprecedented within central nervous system (CNS) development. The anisotropic patterns of progenitor growth obscure earlier dorso‐ventral (Shh‐mediated) patterning. Furthermore, the extensive growth of progenitor population begins to change the overall shape of the hypothalamus, as well as the relative positions of progenitor cells: thus, when first induced, bHyp progenitors directly abut *Foxg1* ^*+*^ *Foxg1^+ive^* telencephalic progenitors but then become displaced by their anterior‐daughters and so, ultimately*, Fgf10* ^*+ive*^ progenitors come to be located in the ventral tuberal hypothalamus (Figure 2).

### 3.4 Molecular mechanisms of basal hypothalamic anisotropic growth

**Figure 3:**
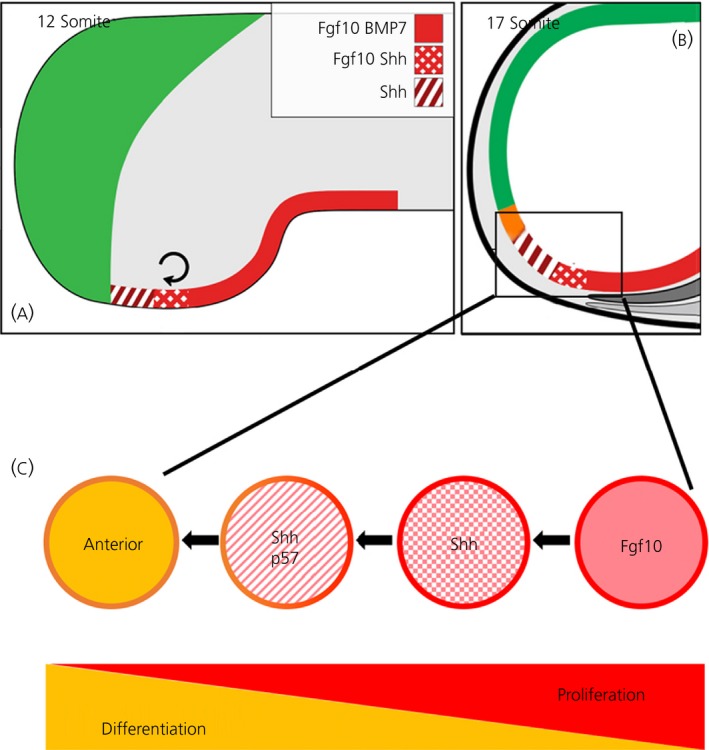
Anterior progenitor growth and differentiation from basal hypothalamic (bHyp) cells. A, Resolution of bHyp cells into posterior *Fgf10/BMP7* ^*+ive*^ (red) and anterior *Fgf10/Shh* ^*+ive*^ (polka dot) domains and onset of growth (depicted by curved arrow). *Shh* ^*+ive*^ neuroepithelial progenitors (hatched) that down‐regulate *Fgf10* grow anteriorly from *Fgf10/Shh* ^*+ive*^ cells. Note that, although shown in sagittal view, *Shh* ^*+ive*^ neuroepithelial progenitors form an arc around *Fgf10* ^*+ive*^ cells. B, Continued generation and differentiation of anterior progenitors (orange) from *Shh* ^*+ive*^ neuroepithelial progenitors. C, A gradient of proliferation and differentiation is detected in the developing hypothalamus, emanating from bHyp progenitors (adapted from Fu et al^1^)

One outstanding question is whether the basal hypothalamus is generated through similar anisotropic sequential growth in other vertebrates. The chick, similar to humans, develops from a flat gastrula, whereas the mouse develops through an egg‐cylinder embryo: potentially, different forces could operate in each, with consequences for hypothalamic progenitor growth. However, studies suggest that, where examined, the molecular mechanisms that lead to anisotropic growth of bHyp progenitors have been conserved across species. In chick, the return to cell cycle and proliferation that drives the development and growth of the basal hypothalamus occurs as the bHyp domain resolves into two *Fgf10* ^*+ive*^ progenitor subtypes: a posterior population that expresses *Fgf10* and *BMP7* (Figure 3A, red) and an anterior population that expresses *Fgf10* and *Shh* (Figure 3A, polka dots). These give rise to progenitor cells that down‐regulate *Fgf10* but retain/up‐regulate Shh and are displaced/migrate anteriorly (Figure 3A, hatched). We term such cells, which derive from bHyp cells, “neuroepithelial *Shh* ^*+ive*^ progenitors”. The mechanism behind the resolution of bHyp cells and generation of neuroepithelial *Shh* ^*+ive*^ progenitors is partly understood and, where investigated, has been conserved: once the transcriptional repressors *Tbx2/Tbx3* are up‐regulated in bHyp cells, they rapidly and directly repress *Shh* by sequestering Sox2 away from a *cis*‐regulatory element in the SBE2 enhancer.^35^, ^43^ Loss of *Shh* is accompanied by the down‐regulation of the Shh receptor, *Patched (Ptc),* in most bHyp cells. However, peripheral bHyp cells behave differently to their central neighbours: they maintain/up‐regulate *Shh* and *Ptc* and down‐regulate *Fgf10* (potentially through loss of the Fgf signalling mediator, pea3).^35^ In this way, the bHyp population quickly gives rise to molecularly‐distinct daughter populations: *Fgf10* ^*+ive*^ cells that overlap with peripheral *Shh* ^*+ive*^ cells. The spatial resolution of *Shh* and *Fgf10* expression is linked to proliferation and depends on the inter‐regulation of, and balance between, Shh and BMP signalling: in chick, if Shh/Shh signalling are aberrantly maintained, proliferation is not stimulated^35^; similarly, mouse Shh^Δhyp^ embryos show a rostral shift in *BMP4* expression^28^ and an expanded zone of non‐proliferating *Tbx3*‐expressing RDVM cells.^29^

### 3.5 Anterior progenitor selection and differentiation

At the same time, a conserved transcriptional programme supports both the selection of anterior neuroepithelial *Shh* ^*+ive*^ progenitors (Figure 2B, hatched) and their subsequent differentiation to anterior progenitors that migrate/are displaced rostrally (Figure 2B,C, orange). Genetic analyses and pharmacological interventions in zebrafish and chick begin to reveal that feed forward‐forward‐back regulatory interactions between Shh and the paired‐box transcription factor Rx (or its zebrafish homologue, rx3) establish a growth loop that selects, and then provides a dynamic stream of anterior neuroepithelial *Shh* ^*+ive*^ progenitors.^8^, ^44^ Conditional genetic analyses indicate that a similar growth loop may exist in mouse,^45^ although this remains to be formally tested. Key to this growth loop is the ability of Shh to non‐autonomously induce *Rx*, for Rx to autonomously induce *Shh*, and for Shh to subsequently autonomously down‐regulate *Rx* for cells to realise the anterior growth programme. Potentially, Shh‐Rx interact additionally with Foxd1 and/or Six3: each interacts with Shh and each promotes proliferation.^33^, ^46^, ^47^, ^48^

Having acted with Rx to select anterior progenitors, in a subsequent step, Shh appears to up‐regulate *p57* ^*kip2*^ 8 and components of the Notch pathway^49^, ^50^ to promote a neurogenic differentiation programme (Figure 2B,C). Up‐regulation of *p57* ^*kip2*^ and Notch components is followed by the up‐regulation of *Shh* itself, through an unknown mechanism. Therefore, in space, there is an opposing differentiation‐proliferation gradient: highest levels of proliferation are detected in *Fgf10* ^*+ive*^ and *Shh* ^*+ive*^ progenitor cells, and highest levels of p57^kip2^ (marking cell cycle exit/differentiation) are detected in daughter cells that have migrated/are displaced furthest away^8^ (Figure 2C).

There are a number of implications to these findings. First, having initially acted as a morphogen to pattern the early hypothalamus, Shh then regulates growth, potentially by regulating the cell cycle. Second, anterior neuroepithelial *Shh* ^*+ive*^ progenitor cells are a dynamic cell population: *Shh* ^*+ive*^ progenitors are constantly being generated. Potentially, this creates a temporal dimension, where waves of progenitors arise in a spatio‐temporal manner from anterior neuroepithelial *Shh* ^*+ive*^ progenitors, providing the opportunity to build complex arrays of basal hypothalamic neurones. In support of this idea, genetic or pharmacological studies that down‐regulate/prevent Shh or Rx activity leads to the failure of differentiation of many different neuronal subtypes of the basal‐anterior and tuberal hypothalamus, including pomc, avp, otp, TH and Sst neurones, and neurones of the tract of the post‐optic commissure.^1^, ^8^, ^18^, ^27^, ^28^, ^29^, ^36^, ^44^, ^45^ However, future lineage‐tracing studies that build on previous/recent studies^8^, ^27^, ^29^, ^51^ are needed to tease out which neurones require Shh non‐autonomously vs autonomously; namely, to distinguish between neurones born from progenitors that respond to Shh (but do not express it) vs neurones that differentiate from *Shh* ^*+ive*^ progenitor populations.

### 3.6 Maintenance of a ventro‐tuberal *Fgf10* ^*+ive*^ progenitor pool

Throughout the generation of anterior neuroepithelial *Shh* ^*+ive*^ progenitors, a pool of *Fgf10* ^*+ive*^ progenitors is established and maintained.^8^ The mechanisms that select a steady supply of anterior neuroepithelial *Shh* ^*+ive*^ progenitors simultaneously form part of the mechanism that ensures a stable‐size pool of *Fgf10* ^*+ive*^ progenitors in a mechanism that may be conserved across species. Thus, genetic and pharmacological interventions suggest that Shh, deriving from anterior neuroepithelial progenitors, feeds back to regulate the size of the *Fgf10* ^*+ive*^ pool^8^, ^36^ (note that Wnt may also be involved^39^, ^40^). Certainly, in other parts of the brain, differentiating cells feedback to progenitor cells to maintain their appropriate numbers and behaviours.^52^ In chick, mouse and zebrafish, progenitor cells within the *Fgf10* ^*+ive*^ pool continue to express Fgf signal components, including pMAPK and pea3, and to respond to juxtacrine Fgf signalling, into late stages of embryogenesis. Their ability to continue to express *Fgf10* and respond to Fgf signalling has important implications for the future development, maintenance and function of the hypothalamus, including formation of the hypothalamic‐pituitary neuraxis (beyond the scope of the present review; Placzek M., Fu T., Towers M. [submitted]).

### 3.7 Mammillary progenitor generation and differentiation

In the short‐term, the maintenance of pools of undifferentiated *Fgf10* ^*+ive*^ progenitor cells is important for generating the mammillary progenitors that begin to appear after anterior progenitors, and that migrate/are displaced posteriorly^8^ (Figure 2C). At present, it is not clear what promotes the switch from anterior to mammillary progenitor generation: indeed, currently, we understand little about either this transition or the process of mammillary progenitor selection/growth, although the transcription factor, Lhx5, plays a role in mammillary differentiation.^53^ Mammillary progenitors appear to be generated from a posterior proliferation front^8^ and alterations in the balance of Shh and BMP signalling disrupt mammillary progenitors and differentiating cells, suggesting some similarities in the programmes of differentiation of anterior and posterior progenitors.^35^, ^41^ Regardless of the mechanism, the extensive growth of anterior then mammillary progenitors obscures earlier patterning. The simple organisation of the hypothalamus along the dorso‐ventral axis, established through early Shh patterning, is rapidly eroded through the subsequent extensive growth of anterior and mammillary progenitor populations.

### 3.8 Infundibular progenitor generation and differentiation

Finally, having generated anterior and mammillary progenitors that extend in opposite directions, the *Fgf10* ^*+ive*^ progenitor pool gives rise to another set(s) of progenitors: infundibular progenitors that grow ventrally.^41^, ^42^, ^54^ Unlike anterior and mammillary progenitors, infundibular progenitors are glial in nature. Potentially, the Notch signalling pathway triggers a switch from neurogenesis to gliogenesis: in *Hes1(−/−)*;* Hes5(+/–)* mutant embryos, progenitor cells differentiate into neurones at the expense of pituicytes (derivatives of the infundibulum: see below).^42^ Other experiments begin to reveal that Fgf10 is itself required for growth of the infundibulum: if *Fgf10* is reduced, eliminated or dysregulated, the infundibulum does not develop and infundibular cells/infundibular‐derived cells are apoptotic and hypoplastic.^8^, ^55^, ^56^ Knockout studies in the mouse and analysis of human variants reveal a number of TFs, such as *Hes1/Hes5*, that are required for infundibular formation, including *Nkx2.1*,* Tbx3* and *Sox2*.^42^, ^57^ Many of these are likely to affect early steps in the development of bHyp progenitors but conditional knockout studies are beginning to show TFs that act downstream of Fgf signalling and underlie the progression or maintenance of glial infundibular progenitors. In particular, Rx and the Lim homeodomain TF, Lhx2, may work downstream of Fgf, and in an inter‐regulatory manner, to specify the infundibulum^45^, ^58^, ^59^, ^60^, ^61^, ^62^: in Lhx2‐deficient mice, the infundibulum fails to grow, cells proliferate aberrantly and show increased cell death.^61^ The SoxB1 HMG‐box transcription factor, Sox3, is likely to interact with Rx/Lhx2: in humans, either reduced or elevated dosage of SOX3 leads to infundibular hypoplasia.^57^

Taken together, then, this sequence of growth leads to progenitor cells of basal anterior, tuberal and mammillary neurones arrayed around the ventro‐tuberal infundibulum. The sequential anisotropic growth in three‐dimensions from bHyp progenitor cells is peculiar and unprecedented within CNS development.

## 4 ANISOTROPIC GROWTH MODEL AND HYPOTHALAMIC ORGANISATION

The “anisotropic growth model” of hypothalamic development shows that, in the chick, different rudiments of the adult hypothalamus are established at different times, suggesting sequential progenitor programmes: an early programme that arises as progenitor cells are born in response to an early GliA‐GliR gradient, followed by a later programme that arises as bHyp progenitor cells develop, and then itself has three temporally‐sequential components: anterior, mammillary and then infundibular. Additionally, the model emphasises the importance of progenitor migration/displacement in establishing different hypothalamic domains, and shows that, in the chick, at least some neurones of the anterior basal hypothalamus are likely to be generated from *Shh*‐expressing progenitor cells (anterior RDVM cells/anterior *Shh* ^*+ive*^ neuroepithelial progenitors). The movement of progenitor cells, whether passively or actively, is likely to occur to a significant extent as the hypothalamus develops over time. Although not examined in detail, alar progenitor cells also undergo extensive migration.^35^ The migration of bHyp and alar progenitor cells explains the difficulty in matching early progenitors to adult neurones and nuclei.

As noted, it remains to be seen whether a similar programme builds the hypothalamus in other species. Current lineage‐tracing studies in the mouse show that *Shh*‐expressing progenitors (either posterior RDVM cells^27^ or *Shh* ^*+ive*^ neuroepithelial cells^29^, ^51^) give rise to tuberal and mammillary regions, although they do not provide evidence that anterior regions are generated from *Shh*‐expressing progenitors. Instead, many existing models suggest that an alar progenitor domain lies between telencephalic and *Shh*‐expressing basal hypothalamic progenitors.^4^, ^5^ One possibility is that different classes of vertebrates have evolved slightly different mechanisms to specify the hypothalamus. An alternate possibility is that current lineage‐tracing studies have not marked anterior‐most RDVM cells or early‐generated *Shh* ^*+ive*^ neuroepithelial cells (the likely sources of anterior progenitors). Certainly, genetic lineage‐tracing studies of progenitor cells support the idea that many mouse hypothalamic cells arise from *Foxd1* ^*+ive*^ progenitor cells that abut telencephalic progenitors.^18^, ^33^

Do gene knockout studies provide insight into how the mouse hypothalamus is built? Previous gene knockout studies have suggested two major transcriptional programmes of hypothalamic development: a Fezf2/Olig2/Otp/Sim1 programme, which generates neurones of the PVN, PeVN, SON and SCN that occupy the anterior hypothalamus, and a Nkx2.1‐Shh‐Rx progenitor programme, which generates neurones of the tuberal hypothalamus, including those of the VMN and ARC; Placzek M., Fu T., Towers M. (submitted).^45^, ^57^, ^62^, ^63^ Thus, for example, initial reports suggested that the PVN/PeVN/SON/SCN, but not the ARC, can be detected in mice that lack functional Nkx2.1,^64^ whereas Lhx1 (a marker of the SCN) is still detected after genetic‐inactivation of *Shh*. Arguably, however, these studies are worth re‐visiting: lineage tracing studies provide evidence for extensive migration in the mouse^65^; indeed, lineage tracing including (a tau‐LacZ knock‐in allele at the Sim1 locus) of mice mutant for Sim1 show that PVN/SON progenitor cells are generated but do not migrate normally.^6^ Thus, Sim1 may direct migration, rather than be a master regulator of lineage. Furthermore, although still present, the PVN/PeVN/SON/SCN appear reduced in size in the Nkx2.1‐null mouse^64^ raising the possibility that neurones within the PVN/PeVN/SON/SCN may be composed of mixed progenitor origin: some arising from bHyp cells via an Nkx2.1‐Shh‐Rx programme, and some arising from displaced/migrated alar progenitors via a Fezf2/Olig2/Otp/Sim1 programme.

In summary, the final position of hypothalamic neurones does not necessarily reflect the position of their progenitors, which can migrate extensively. This highlights the importance of future lineage‐tracing studies in determining the origin of individual hypothalamic neuronal classes in discrete nuclei.
